# The mediating role of psychological flexibility in the relationship between self-efficacy and loneliness in young adulthood

**DOI:** 10.3389/fpsyg.2026.1878259

**Published:** 2026-08-03

**Authors:** Mustafa Kumru, Tuğba Seda Çolak Turan, Betül Düşünceli

**Affiliations:** 1 Esenyurt Guidance and Research Center, Ministry of National Education, Istanbul, Türkiye; 2 Department of Educational Sciences, Faculty of Education, Psychological Counseling and Guidance, Düzce University, Düzce, Türkiye; 3 Department of Educational Sciences, Faculty of Education, Psychological Counseling and Guidance, Sakarya University, Sakarya, Türkiye

**Keywords:** loneliness, mediation, psychological flexibility, self-efficacy, young adulthood

## Abstract

**Inroduction:**

Loneliness is a common and universal human experience associated with adverse psychological outcomes. Therefore, identifying psychological factors that reduce loneliness and strengthen individuals’ coping capacity may contribute to the development of more effective psychological interventions.

**Methods:**

The primary aim of this study was to examine the associations among self-efficacy, psychological flexibility, and loneliness, with particular emphasis on the indirect association between self-efficacy and perceived loneliness through psychological flexibility in a cross-sectional model. Data were collected using a Personal Information Form, the SELSA-S Social and Emotional Loneliness Scale, the Psychological Flexibility Scale, and the Self-Efficacy Scale. Pearson product-moment correlation analysis was conducted to examine the associations among the variables, and regression analysis was performed to examine the associations between self-efficacy, perceived loneliness, and psychological flexibility. The bootstrapping mediation analysis using the PROCESS macro (Model 4, 5000 bootstrap resamples) was conducted to examine the indirect association between self-efficacy and perceived loneliness through psychological flexibility. The sample consisted of 412 young adults recruited through convenience sampling.

**Results:**

The findings indicated that self-efficacy was positively associated with psychological flexibility and negatively associated with perceived loneliness, whereas psychological flexibility was negatively associated with perceived loneliness. Furthermore, a statistically significant indirect association between self-efficacy and perceived loneliness through psychological flexibility was observed in the cross-sectional model, whereas the direct association was not statistically significant.

**Discussion:**

These findings suggest that psychological flexibility may be relevant to the association between self-efficacy and perceived loneliness. However, given the cross-sectional design and convenience sampling, the findings should not be interpreted as evidence of causal mediation.

## 1 Introduction

Humans as social beings need a safe social environment and relationships to survive. Deterioration of social relationships negatively impacts the healthy life in many ways, leading to loneliness in particular (Mushtaq et al., 2014). Being a universal human experience (Bekhet et al., 2008), loneliness is among the most common problems (Weiss, 1975) and a vast source of anxiety for both the elderly (Mullins and McNicholas, 1986) and adults (Yanguas et al., 2018). Especially for adolescence and young adulthood loneliness considered as a global public mental health problem (Hemberg et al., 2022). Loneliness was explained as a relational deficiency (Zack, 1985). Loneliness is described as a complex set of emotions that can hinder social development and negatively impact physical and mental health (Ernst and Cacioppo, 1999; Krause-Parello, 2008). In Cacioppo and associates’ studies, loneliness is associated with various individual differences such as the feeling of vulnerability, anxiety, anger, negative state of mind, fear of negative evaluation, weak social skills, low social support, discouragement, misfit (Cacioppo et al., 2006; Hawkley and Cacioppo, 2010), depression, social withdrawal, enmity, alienation, pessimism, low positive affect, and shyness (Ernst and Cacioppo, 1999). It is emphasized that loneliness is an important risk factor for psychological disorders (Cacioppo et al., 2000) and can lead to various problems such as depression, alcohol addiction, sleep problems, personality disorders, and Alzheimer’s (Mushtaq et al., 2014). Considering this, compared to those who do not suffer from loneliness, lonely individuals are expected to have higher negative affect levels, manifesting introverted behaviors in social life, feeling distrust towards others and themselves, having little control over success or failure, and showing less satisfaction with their relationships. Loneliness is believed to be influenced by both genetic and environmental factors (Cacioppo et al., 2006). Three intervention methods, such as awareness to reduce loneliness with the consideration of related roots, social-cognitive training, and social support, are emphasized (Veronese et al., 2021). It is stated that loneliness can be less painful as self-efficacy in particular increases (Killeen, 1998). At this point, exploring the factors affecting loneliness is thought to have a positive effect on gaining an awareness of loneliness.

### 1.1 Self-efficacy and loneliness

One of the important concepts affecting loneliness is thought to be self-efficacy. Self-efficacy is commonly considered a key construct in motivation and performance research. It refers to individuals’ beliefs in their capability to successfully complete tasks and manage specific demands (Mathews, 2005). This belief has a multifaceted developmental network fed by four main factors: performance success, vicarious experience, verbal persuasion, and physiological states (Bandura, 1977). Likewise, it functions as a multilevel and multifaceted set of beliefs that affect how people will feel and think, how they will motivate themselves, and how they will behave in different situations (Tsang et al., 2012). It is stated that self-efficacy belief has a greater impact on people’s motivations, emotions and actions than reality and objective truths, especially in areas such as the way people think about themselves and others, their behavioral choices, their determination to pursue goals, their level of perseverance, their resilience to setbacks and problems, and their stress levels (Bandura, 1997). Self-efficacy is expressed as a critical factor that is related to many areas such as cognitive and intellectual functioning, health, clinical problems such as anxiety, phobias, depression, eating disorders, alcohol problems, and drug addiction, as well as to athletics and exercise activity, organizations, politics, and social change (Bandura, 1997).

Self-efficacy refers to the belief in one’s competence and is believed to affect social interactions (Kumar and Pavithra, 2012). Individuals with low self-efficacy perception have low tolerance and resilience against negative emotions, have difficulty in balancing emotions and behavior (Altıntaş, 2015), cannot express themselves comfortably and avoid interpersonal relationships (Parçal, 2018), and prefer to stay in the background and remain passive (Noyan Yalman and Özkaynar, 2018) become more vulnerable to stress and depression, avoid difficult tasks with the perception of failure, act with perceptions of inadequacy rather than perceptions of control in line with their goals, and thus lose their self-belief and confidence (Bandura, 1994). In this respect, it is stated that the concept of self-efficacy plays a critical role in self-improvement, change, and adaptation (Bandura, 2012a,b), increasing psychological resilience (Liu et al., 2018), and feeling free (Bandura, 1997). It is emphasized that it is closely related to factors such as learning skills (Zimmerman, 2000), capacity to cope with problems (Freudenberg et al., 2010), and psychological well-being level (Gülşen, 2013).

### 1.2 Psychological flexibility and loneliness

Another concept thought to be related to loneliness and to have a significant effect on loneliness is psychological flexibility. Psychological flexibility, which is a central concept for self-awareness (Palladino et al., 2013) and refers to both internal and interpersonal processes, has an important place in human life (Gloster et al., 2017). Psychological flexibility encompasses a wide range of human abilities such as recognizing and adapting to various situational requirements; changing thoughts or behavioral repertoires when the strategies used compromise personal or social functioning; balancing between different life domains; and awareness of behaviors that are compatible with ingrained values (Gloster et al., 2011; Kashdan and Rottenberg, 2010).

It is stated that psychological flexibility can be increased through the development of acceptance and value-based action skills (Fledderus et al., 2010). As a result, psychological flexibility consists of cognitive defusion, acceptance, contextual self, staying in the moment, and taking action in line with values (Levin et al., 2012). In this respect, psychological flexibility provides the ability to act in accordance with values in the presence of internal disturbances such as pain and distress (Faulkner et al., 2020; Ong et al., 2019). Psychological flexibility has a positive effect on life satisfaction (Lucas and Moore, 2020) and psychological well-being (Guerrini Usubini et al., 2021); it has a significant effect on chronic pain, anxiety (Gentili et al., 2019), and depression, and as a result, it has been found to reduce the accumulated negative impact of significant life events (Fledderus et al., 2013; Fonseca et al., 2020). Poor psychological flexibility is associated with clinical problems as well as daily life difficulties, and efforts to increase psychological flexibility have been associated with improvement in various psychological difficulties, including substance abuse (Luoma et al., 2011) and chronic pain (McCracken and Velleman, 2010). Psychological flexibility was found to be negatively associated with anxiety, searching for meaning, avoiding emotions, feeling burnout, alexithymia, denial behavior, rumination, experiencing test anxiety, intolerance to uncertainty, Covid-19 anxiety, positively associated with the existence of meaning in life, perception of competence, marital adjustment, occupational satisfaction, psychological well-being, authenticity, life satisfaction, sleep quality, and the ability to cope with problems (Acar, 2022; Ulubay and Güven, 2022).

Psychological flexibility is thought to increase individuals’ resilience against difficulties and increase their tolerance against negativities in terms of enabling them to cope with life challenges in a healthy way. Similarly, the understanding of acceptance within psychological flexibility is expected to increase individuals’ adaptability to change. Considering the frequency and intensity of change and transformation processes in human life, adaptation skills are considered to be of critical importance. However, there is an expectation that incompatibility in change processes may lead the individual to loneliness. It is stated that change-oriented factors such as age, living alone, widowhood, low education level, low income level, poor health status, decreased functionality, poor vision and hearing, and low quality of social relationships are factors associated with loneliness (Cohen-Mansfield et al., 2016; Savikko et al., 2005). This suggests that loneliness is in contact with many areas of life in terms of both cause and effect.

### 1.3 Relations between loneliness, self-efficacy and psychological flexibility

A growing body of research has examined the association between self-efficacy and loneliness. Recent studies consistently indicate that higher levels of self-efficacy are associated with lower levels of perceived loneliness (Odacı et al., 2025; Patel and Ojha, 2025; Szcześniak et al., 2025). Beyond this direct association, self-efficacy has been linked to greater emotional resilience and more adaptive coping with negative emotional experiences (Odacı et al., 2025). In addition, Szcześniak et al. (2025) reported that adolescents with higher self-efficacy experienced fewer difficulties in peer communication. These findings suggest that self-efficacy may facilitate adaptive psychological processes that help individuals maintain interpersonal relationships and cope more effectively with emotionally challenging situations.

Recent studies have increasingly highlighted the role of psychological flexibility in understanding loneliness. For example, Jormanainen et al. (2026) reported that psychological flexibility moderated the association between social withdrawal and loneliness and identified lower psychological flexibility as a risk factor for increased loneliness during the transition to adolescence. Similarly, Vaca-Gallegos et al. (2025) found that psychological flexibility was associated with lower perceived stress, which in turn was associated with lower levels of loneliness. Tilburg et al. (2024) further demonstrated that psychological flexibility moderated the relationship between loneliness and mental health, such that higher levels of psychological flexibility attenuated the negative impact of loneliness on mental health outcomes. Consistent with these findings, Sevinawati and Orizia (2023) reported that psychological inflexibility was positively associated with loneliness among young adults. Taken together, these findings suggest that psychological flexibility is consistently associated with lower loneliness and may represent an important psychological mechanism underlying this relationship. Individuals with greater psychological flexibility may be better able to accept difficult emotions and remain engaged in valued social behaviors despite experiencing distress. Therefore, psychological flexibility may help explain how personal psychological resources, such as self-efficacy, are associated with lower levels of perceived loneliness.

Psychological flexibility and self-efficacy have been found to be positively related (Mursalzade et al., 2025; Razazan, 2025; Wang et al., 2025). However, there is a gap in the literature regarding the role of psychological flexibility in the relationship between self-efficacy and loneliness. Psychological flexibility reflects the capacity to remain engaged in valued actions while accepting difficult thoughts and emotions. From this perspective, it may represent a potential mechanism through which self-efficacy is associated with lower perceived loneliness. Individuals with higher self-efficacy may be more likely to approach social challenges, adapt to stressful interpersonal situations, and remain psychologically flexible rather than withdraw from social interactions. Consequently, psychological flexibility may help explain the association between self-efficacy and perceived loneliness.

Consequently, it is hypothesized that self-efficacy and psychological flexibility are associated with perceived loneliness. In line with this assumption, the present study aims to examine the associations of self-efficacy and psychological flexibility with perceived loneliness. Accordingly, the following research hypotheses were formulated.

*H_1_*: There is a positive correlation between individuals’ self-efficacy and psychological flexibility.

*H_2_*: There is a negative correlation between individuals’ self-efficacy and loneliness perceptions.

*H*_3_: There is a negative correlation between individuals’ psychological flexibility and loneliness perceptions.

*H_4_*: Psychological flexibility has a mediating role in the effect of individuals’ self-efficacy on their perceptions of loneliness.

## 2 Methods

In this study, the aim was to examine the mediating role of psychological flexibility in the effect of self-efficacy on loneliness perception. In this context, the relational survey model was used. This model explains the level of relationship between more than one variable, as well as the predictive power of variables on each other (Creswell, 2014).

### 2.1 Sample

The population of the study consists of adults aged 18 years and over, and the sample consists of 466 participants selected from the population by a convenience sampling method. Data were collected online from a nationwide sample in Türkiye between February 1, 2023, and November 1, 2023. The inclusion criteria for the study required participants to be between 18 and 30 years of age; individuals outside this age range were excluded from the study. Although a total of 466 responses were initially obtained, those meeting the exclusion criteria were removed from the dataset, resulting in a final sample size of 412 participants for the data analysis. Of the 412 participants, 280 were female and 132 were male. In terms of the economic status variable, 67 participants had low, 330 participants had medium, and 15 participants had high economic status.

### 2.2 Procedure

The procedures within the scope of the current study were carried out in accordance with ethical standards, observing the confidentiality of personal data, and with the participation of voluntary participants. In this context, ethics committee permission dated 27.04.2023 and decision number 2023/91 was obtained from Düzce University Scientific Research and Publication Ethics Committee. The anonymized dataset supporting the findings of this study is available in the Open Science Framework (OSF) repository: https://osf.io/k93wm/files/w2yj4.

Statistical analyses were performed using IBM SPSS Statistics 23.0 along with the PROCESS macro (Version 4.2; Hayes, 2022). Prior to the main analyses, parametric test assumptions were rigorously evaluated (Table 1). Data normality was confirmed as skewness and kurtosis values fell within the −1.5 to +1.5 range (Tabachnick and Fidell, 2013). Multicollinearity was ruled out since Variance Inflation Factor (VIF) values were below 5 and Tolerance values were above 0.10 (Hair et al., 2010). The Durbin-Watson statistic (1.72) confirmed the assumption of independent errors, as it fell within the ideal range of 1.5 to 2.5 (Field, 2009). Additionally, regression diagnostics using Cook’s distance (Maximum = 0.04) indicated no influential outliers or distorting cases capable of biasing the regression models. To examine the associations among the study variables, Pearson product–moment correlation analyses were conducted. The proposed indirect association was examined using an ordinary least squares (OLS)-based mediation analysis (PROCESS Model 4) with 5,000 bootstrap resamples and 95% bias-corrected confidence intervals. Age, gender, and perceived economic status were included as covariates in the mediation model. An indirect association was considered statistically significant when the 95% bootstrap confidence interval did not include zero. Given the cross-sectional design, the mediation analysis was interpreted as a statistical model of indirect association rather than evidence of causal mediation.

**Table 1:** Descriptive statistics.

	*N*	Range	Minimum	Maximum	Sum	Mean	Std. Deviation	Variance	Skewness	Kurtosis	Reliability coefficient
Psychological Flexibility	412	79.00	15.00	94.00	19925.00	48.3617	16.27180	264.772	0.081	−0.184	0.799
Self-Efficacy	412	115.00	61.00	176.00	50970.00	123.7136	19.83682	393.499	0.069	0.115	0.943
Loneliness	412	106.00	59.00	165.00	51315.00	124.5510	22.43921	503.518	−0.270	−0.506	0.815

### 2.3 Instrument

The data were collected through the Personal Information Form, SELSA-S Social and Emotional Loneliness Scale, Psychological Flexibility Scale, and Self-Efficacy Scale. The Personal Information Form was prepared by the researchers.

*SELSA-S Social and Emotional Loneliness Scale* is a short form of the original SELSA scale. It was developed by DiTommaso et al. (2004) and adapted to Turkish by Akgül (2020). This scale, which was created using a 7-point Likert-type rating scale, consists of 15 items. The Cronbach’s alpha reliability coefficient of the scale was calculated as 0.83 (Akgül, 2020). In the present study, Cronbach alpha was 0.81.

*The Psychological Flexibility Scale* was developed by Francis et al. (2016) and adapted into Turkish by Karakuş and Akbay (2020). It is a 7-point Likert-type rating scale consisting of 28 items. The Cronbach Alpha reliability coefficient of the scale was calculated as 0.79 (Karakuş and Akbay, 2020). In the present study, Cronbach alpha was 0.79.

*The Self-Efficacy Scale,* developed by Akın (2007), aims to determine the self-confidence/self-efficacy levels of individuals. Akın (2007) conceptualized self-confidence within the framework of self-efficacy. This 5-point Likert-type scale consists of 33 items. The Cronbach’s alpha reliability coefficient of the scale was calculated as 0.83 (Akın, 2007). In the present study, Cronbach alpha was 0.94.

For all scales, total scores were calculated by summing the item scores after reverse-scored items had been recoded where applicable. Because the questionnaires were administered online and all items were set as mandatory responses, no missing data occurred at the item level.

## 3 Results

Pearson Correlation Analysis was applied to determine the relationship between self-efficacy, psychological flexibility, and loneliness perception. The findings of the analysis are shown in Table 2. As a result of the analysis, it was found that there was a moderate positive and statistically significant relationship between psychological flexibility and self-efficacy [*r* = 0.615, *p* < 0.01, (0.55, 0.67)]. There was a moderate negative and statistically significant relationship between loneliness perception and psychological flexibility [*r* = −0.511, *p* < 0.01, (−0.58, −0.42)]. There was also a statistically significant moderate negative relationship between perception of loneliness and self-efficacy [*r* = −0.360, *p* < 0.01, (−0.44, −0.27)] (Table 2).

**Table 2:** Pearson correlations among study variables.

	1	2	3
1. Psychological flexibility	1		
2. Self-Efficacy	0.615**	1	
3. Loneliness	−0.511**	−0.360**	1

**p < 0.01.

Simple Linear Regression Analysis was conducted to determine the relationship between psychological flexibility, perception of loneliness, and self-efficacy. The regression analysis findings regarding self-efficacy and psychological flexibility are shown in Table 3. As a result of the analysis, it was seen that self-efficacy was positively associated with psychological flexibility (*β* = 0.615, *t* = 15.78, *p* < 0.001). Self-efficacy explained 37.8% of the change in psychological flexibility (*R^2^* = 0.378) (Table 3).

**Table 3:** Regression analysis predicting psychological flexibility from self-efficacy.

Independent variable: self-efficacy							
Dependent variable	*R* ^2^	Adjusted *R*^2^	*B*	Beta (*β*)	*t*	*F*	*p*
Psychological flexibility	0.378	0.376	0.543	0.615	15.780	249.015	0.000

Note 3: The regression analysis findings regarding psychological flexibility and loneliness perception are shown in Table 4. As a result of the analysis, it was observed that psychological flexibility was negatively associated with perceived loneliness (*β* = −0.419, *t* = −12.032, *p* < 0.01). Psychological flexibility explained 26.1% of the change in loneliness perception (*R^2^* = 0.261) (Table 4).

**Table 4:** Regression analysis predicting loneliness from psychological flexibility.

Independent variable: psychological flexibility							
Dependent variable	*R* ^2^	Adjusted *R*^2^	*B*	Beta (*β*)	*t*	*F*	*p*
Loneliness	0.261	0.259	−0.419	−0.511	−12.032	144.759	0.000

Note 4: The regression analysis findings regarding self-efficacy and loneliness perception are shown in Table 5. As a result of the analysis, it was observed that self-efficacy was negatively associated with loneliness perception (*β* = −0.360, *t* = −7.822, *p* < 0.01). Self-efficacy explained 13% of the change in loneliness perception (*R^2^* = 0.13) (Table 5).

**Table 5:** Regression analysis predicting loneliness from self-efficacy.

Independent variable: self-efficacy							
Dependent variable	*R* ^2^	Adjusted *R*^2^	*B*	Beta (*β*)	*t*	*F*	*p*
Loneliness	0.130	0.128	−0.261	−0.360	−7.822	61.188	0.000

**Figure 1:**
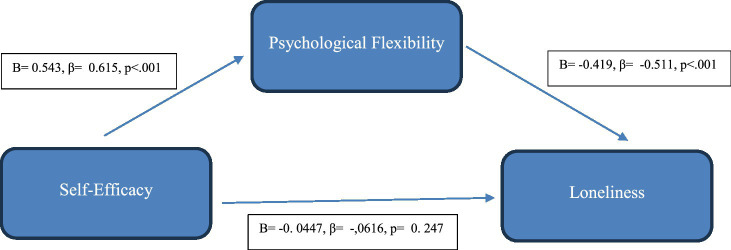
The mediating role of psychological flexibility in the relationship between self-efficacy and loneliness among young adults.

Note 5: A bootstrap regression analysis using PROCESS Model 4 with 5,000 bootstrap resamples was conducted to examine the indirect association between self-efficacy and perceived loneliness through psychological flexibility while controlling for age, gender, and perceived economic status (Table 6). Results indicated that the total association between self-efficacy and perceived loneliness was statistically significant [*b* = −0.24, 95% CI (−0.30, −0.17)]. After psychological flexibility was included in the model, the direct association was no longer statistically significant [*b* = −0.04, 95% CI (−0.12, 0.03)]. The bootstrap analysis indicated a statistically significant indirect association through psychological flexibility [*b* = −0.19, 95% Boot CI (−0.25, −0.14)]. Given the cross-sectional design (Figure 1), this indirect association should not be interpreted as evidence of a causal mediation process.

**Table 6:** Bootstrap regression analysis results (*N* = 412).

Variables	Effect (b)	Beta *(β)*	*SE*	*t*	*p*	LLCI	ULCI
The total effect of X on Y	−0.2377	−0.3277	0.0332	−7.1637	0.000	−0.3029	−0.1724
Age	1.1948	0.0343	1.5776	0.7574	0.4493	−1.9064	4.2961
Gender	−5.8847	−0.1129	2.3581	−2.4955	0.0130	−10.5203	−1.2490
Economic Status	−6.5558	−0.1726	1.7368	−3.7747	0.0002	−9.9700	−3.1416
The direct effect of X on Y	−0.0447	−0.0616	0.0386	−1.1572	0.2.479	−0.1206	0.0312

	Effect (b)	Bootstrap *SE*	BootLLCI	BootULCI
The indirect effect of X on Y through M	−0.1930	0.0267	−0.2460	−0.1429

Self-efficacy (X), loneliness (Y), and psychological flexibility (M).

Age, gender, and perceived economic status were included as covariates in the mediation model.

## 4 Discussion

### 4.1 The findings of the present study are discussed in relation to the existing literature

In the current study, it was observed that there was a moderate positive relationship between self-efficacy and psychological flexibility and that self-efficacy was related to psychological flexibility positively. In other words, as individuals’ self-efficacy levels is associated with higher levels of their psychological flexibility. These findings are consistent with previous studies in the literature (Jeffords et al., 2020; Wang and Zhang, 2021). Self-efficacy is closely related to self-knowledge, acceptance of one’s characteristics, and confidence in one’s abilities (McKay and Fanning, 2016; Schunk and Pajares, 2002). Bandura (1977) emphasized that such beliefs are related to performance achievements and social experiences. Emotional factors such as acceptance, cognitive factors such as beliefs, and behavioral factors such as performance achievements, which are associated with self-efficacy, also reflect the basic concepts of psychological flexibility. At this point, it is thought that a strong reason for the relationship between self-efficacy and psychological flexibility is that they rooted in shared underlying factors. People with high self-efficacy are likely to more resilient against external psychological and emotional threats because they know themselves better and can accept themselves more easily. They will feel less need to defend themselves against perceptual threats because they have fewer doubts about themselves. Therefore, self-efficacy enables the individual to move away from the psychological rigidity that causes the individual to be in a state of defense. On the other hand, it expands the power and dominance area of psychological flexibility, which enables the individual to regulate himself or herself according to time, event, situation, and person. At this point, Bandura (2012a,b) states that the higher the self-efficacy, the more the decision options and the interest in these options will increase, so that the individual will feel more ready at the point of development and will stand stronger against the obstacles on this path.

According to another finding of the current study, there is a negative moderate relationship between psychological flexibility and loneliness perception, and psychological flexibility was related to loneliness perception negatively. According to these findings, it is seen that individuals with low levels of psychological flexibility have a higher perceived sense of loneliness. The studies in the relevant literature support the current findings (Frinking et al., 2020; Indra et al., 2021; Kelly et al., 2022). Psychological flexibility enhances the individual’s ability to adapt to the time, situation, and social environment (Daşcı et al., 2023; Flujas-Contreras et al., 2023). Adaptability serves as a fundamental requirement for people to live together. It is expected that individuals with poor adaptation skills will first feel lonely, excluded and then gradually become socially isolated. The perception of loneliness involves an internal experience before being an external phenomenon. It is thought that the perception of loneliness is closely related to the past experiences of the individual and is shaped by emotional traces, judgments, and beliefs carried from these experiences. In particular, it is expected that core beliefs of lovelessness and worthlessness may be linked to the individual to the perception of loneliness. At this point, it is seen that factors such as having difficulty in accepting emotions, having rigid thoughts, being firmly attached to thoughts, and having a conceptual and labeling attitude contribute to the perception of loneliness. These factors also appear as factors that explain psychological rigidity, which is the opposite of psychological flexibility (Harris, 2009). Therefore, it is expected that individuals with low levels of psychological flexibility will be more psychologically rigid and feel a stronger sense of loneliness.

According to another finding in the current study, it was observed that there was a negative moderate relationship between self-efficacy and loneliness perception, and that self-efficacy was related to loneliness perception negatively. In other words, it was observed that individuals with high self-efficacy had lower perceptions of loneliness. The studies in the literature support the current findings (Aydın et al., 2018; Dussault and Deaudelin, 2001; Erozkan and Deniz, 2012; Kaplan et al., 2019; Larasati Yunanto et al., 2021; Mercan et al., 2015; Yaman, 2022). The relationship between self-efficacy and loneliness is thought to be related to the closeness of both concepts to social experience. In fact, the result that supportive social experiences improve self-efficacy, while social experiences involving judgment and criticism weaken self-efficacy, is evident in scientific studies and in real life. It is stated that the perception of loneliness emerges as a result of a lack of relationships and social experience (Zack, 1985). Cantor (2020) explains loneliness as a subjective feeling affected by the inadequacies perceived in one’s relationships with others. In support of this, Şahan and Ceyhan (2019) found that social competence affects loneliness. Self-efficacy may be associated with a more positive self-view. It may also be associated with reduced perceived dependence on others, as individuals who perceive themselves as competent are more likely to engage in healthier social relationships. As a result, the perception of loneliness will weaken with the effect of both the sense of efficacy and social support. Self-efficacy is significantly affected by social emotions such as guilt and shame (Kumru, 2022). Feelings of guilt and shame, which are closely related to early experiences, cause the individual to feel faulty and defective and, as a result, to feel excluded. Being faulty and defective means being socially out of the norm. Thinking that one is different from the norm or feeling excluded is thought to be at the center of the perception of loneliness.

Consistent with the main aim of the study, the findings indicated a statistically significant indirect association between self-efficacy and perceived loneliness through psychological flexibility in the present cross-sectional model. This suggests that the relationship between self-efficacy and loneliness may be explained, in part, through psychological flexibility. However, given the cross-sectional correlational design of the study, these results should be interpreted as an indirect association rather than evidence of causal mediation. Accordingly, it is seen that the level of psychological flexibility is associated with the perception of loneliness independently of self-efficacy. At this point, it is thought that the factors related to self-efficacy, which are shaped by psychological flexibility, especially the negative core beliefs of worthlessness and lovelessness, negative social emotions such as guilt and shame, and the avoidance and passivity behaviors that emerge with them, are the factors affecting the perception of loneliness. In other words, it is suggested that the association between self-efficacy and the perception of loneliness may be explained by the underlying mechanisms through which psychological flexibility is associated with self-efficacy. In support of this, Rizzo and Schwartz (2021) found that psychological flexibility predict self-efficacy. In another study, it was observed that individuals with high psychological flexibility had high self-efficacy, while individuals with low psychological flexibility had low self-efficacy (Jeffords et al., 2020). In this context, psychological flexibility is expected to enhance self-efficacy, by addressing negative automatic thoughts and maladaptive core beliefs through cognitive defusion, regulating guilt and shame through emotional acceptance, and reducing avoidance and passivity through value-oriented actions. Indeed, psychological flexibility is considered a significant factor in the positive effects of self-efficacy on coping with problems, mental health, and overall well-being (Schéle et al., 2021).

On the other hand, psychological flexibility has been shown to play a critical role in the perception and experience of loneliness (Frinking et al., 2020). Individuals with lower psychological flexibility tend to be less resilient and more susceptible to feelings of loneliness (Indra et al., 2021). Furthermore, the impact of stressors is likely to increase (Ortega-Jiménez et al., 2021), particularly as cognitive flexibility declines with aging and reduced capacities, resulting in more intense experiences of loneliness (Tohidifar et al., 2021). Within this framework, psychological flexibility can buffer the effects of stress (Arslan and Allen, 2022), reduce the perception of loneliness, and act as a protective factor for mental health (Constantinou et al., 2021; Holly, 2020).

In conclusion, a positive and statistically significant correlation was found between self-efficacy and psychological flexibility whereas the correlation between self-efficacy and loneliness was negative and statistically significant. Similarly, a negative and statistically significant correlation was found between psychological flexibility and loneliness. Based on these findings, H_1_, H_2_, and H_3_ hypotheses of the study were supported. The hypothesis (H_4_), “Psychological flexibility has a mediating role in the effect of individuals’ self-efficacy on their perceptions of loneliness,” was also supported by the study results. The observed association between psychological flexibility and loneliness suggests that psychological flexibility may be a relevant factor to consider in future research on loneliness. Intervention studies are needed to determine whether enhancing psychological flexibility can contribute to coping with loneliness. Practices in clinical and educational settings may benefit from considering the relationship between these variables in efforts to address loneliness and its negative consequences. In this cross-sectional convenience sample, self-efficacy was positively associated with psychological flexibility and negatively associated with loneliness. Psychological flexibility was also negatively associated with loneliness. A bootstrap indirect effect was statistically significant, suggesting that the association between self-efficacy and loneliness may partly operate through psychological flexibility. However, because all variables were measured at a single time point using self-report questionnaires, the findings should not be interpreted as evidence of causal mediation.

### 4.2 Limitations and future directions

Several limitations should be considered when interpreting the findings of this study. First, participants were recruited through convenience sampling, which may limit the representativeness of the sample and the generalizability of the findings to broader populations. Second, the sample predominantly consisted of female participants. Therefore, the observed relationships may not fully reflect patterns present in more gender-balanced populations. Third, the cross-sectional correlational design precludes conclusions regarding the directionality or causality of the observed associations. Longitudinal and experimental studies are needed to examine temporal and causal relationships among the variables. Finally, all data were collected through self-report measures, which may be subject to common method bias, social desirability effects, and reporting inaccuracies. These factors may be associated with the variables examined in the present study and could influence the observed relationships. Future research should consider incorporating such variables to provide a more comprehensive understanding of the findings.

Building on the findings and limitations of the present study, several directions for future research can be proposed. First, longitudinal studies are needed to examine the development of these variables over time and to investigate potential causal and reciprocal relationships among them. Such studies may help clarify the temporal ordering of the observed associations. Second, experimental intervention studies could directly evaluate whether programs designed to enhance psychological flexibility and self-efficacy are effective in reducing perceived loneliness. Such research would provide valuable evidence regarding the potential of these variables as intervention targets. Third, although the present mediation model statistically controlled for age, gender, and perceived economic status, other potentially relevant variables, such as relationship status, mental health history, and perceived social support, were not included. Future studies should incorporate these factors to provide a more comprehensive understanding of the associations observed in the present study. Finally, cross-cultural research is needed to examine whether the proposed model is generalizable across different cultural contexts, thereby contributing to a broader understanding of the psychological processes underlying self-efficacy, psychological flexibility, and perceived loneliness.

## Data Availability

The anonymized dataset supporting the findings of this study is available in the Open Science Framework (OSF) repository at https://osf.io/k93wm/files/w2yj4.
